# Third molar eruption in dental panoramic radiographs as a feature for forensic age assessment – new reference data from a German population

**DOI:** 10.1186/s13005-024-00431-3

**Published:** 2024-05-10

**Authors:** Maximilian Timme, Jan Viktorov, Laurin Steffens, Adam Streeter, André Karch, Andreas Schmeling

**Affiliations:** 1 Institute of Legal Medicine, University Hospital Münster, Röntgenstraße 23, 48149 Münster, Germany; 2 Institute of Epidemiology and Social Medicine, University of Münster, Domagkstraße 3, 48149 Münster, Germany

**Keywords:** Age estimation, Dental age, Wisdom tooth, Orthopantomogram, Chronological age

## Abstract

Forensic age assessment in the living can provide legal certainty when an individual’s chronological age is unknown or when age-related information is questionable. An established method involves assessing the eruption of mandibular third molars through dental panoramic radiographs (PAN). In age assessment procedures, the respective findings are compared to reference data. The objective of this study was to generate new reference data in line with the required standards for mandibular third molar eruption within a German population. For this purpose, 605 PANs from 302 females and 303 males aged 15.04 to 25.99 years were examined. The PANs were acquired between 2013 and 2020, and the development of the mandibular third molars was rated independently by two experienced examiners using the Olze et al. staging scale from 2012. In case of disagreement in the assigned ratings, a consensus was reached through arbitration. While the mean, median and minimum ages were observed to increase with each stage of mandibular third molar eruption according to the Olze method, there was considerable overlap in the distribution of age between the stages. The minimum age for stage D, which corresponds to complete tooth eruption, was 16.1 years for females and 17.1 years for males. Thus, the completion of mandibular third molar eruption was found in both sexes before reaching the age of 18. In all individuals who had at least one tooth with completed eruption and who were younger than 17.4 years of age (*n* = 10), mineralization of the teeth in question was not complete. Based on our findings, the feature of assessing mandibular third molar eruption in PAN cannot be relied upon for determining age of majority.

## Introduction

The eruption of teeth is characterized as the process by which teeth transition from their initial developmental location within the osseous jaw to their functional position within the oral cavity [1]. Tooth eruption is primarily governed by genetic factors, with contributions from the local environment [1]. The eruption process has been divided into five distinct stages for convenience: the pre-eruptive stage, the intra-osseous stage, the mucosal penetration stage, the pre-occlusal stage, and the post-occlusal stage [2].

Due to the primary genetic control of eruption, the temporal sequence of the stages is replicable, and the temporal variability in the course of the stages is relatively low. This makes it, in principle, possible to infer the chronological age of the person from the status of the eruption, in the sense of tooth age. Nowadays, the term “dental age estimation” (DAE) has come into use for such procedures, which includes other age-related dental traits in addition to eruption [3–5]. According to reports, DEA has not only been consulted since the beginning of modern times but has been used throughout history. In ancient Rome, the minimum age for military service was determined by assessing the eruption of specific teeth into the oral cavity [6]. However, tooth eruption is by no means entirely devoid of external factors, despite its primarily genetic control. Genetic variations, for instance, within the context of specific syndromes, can impact tooth eruption [1]. Furthermore, other external factors, such as those that might obstruct the eruption path of a tooth, can slow down this process or stop it completely [7]. In view of the correlation of eruption and chronological age, it becomes obvious that teeth affected in this way do not allow conclusions to be drawn from about chronological age and therefore must not be used for DAE.

Today, forensic age assessment needs be conducted whenever a person’s age is uncertain or when legitimate doubts exist regarding the provided age information [8]. These cases have recently become more frequent, driven by increasing cross-border migration [8, 9]. As a result, forensic age assessment has recently become a focal point within the field of forensic science [10–18]. Notably, the principle of DAE remains an integral component of relevant recommendations for evidence-based age assessment procedures to this day [19]. In accordance with recommendations for age assessment, this should encompass, among other investigations, a radiographic examination of the dentition [20]. Since in many legal proceedings today the question of the possible majority of the person is the central issue [8], evaluation is usually based on the third molars, since the development of these teeth is the last to be completed and the age of majority falls in the period in which the development of the third molars comes to an end [21]. Specifically, the mineralization and eruption of the mandibular third molars are assessed [8]. The findings are then compared with corresponding reference populations to estimate the chronological age of the individual, with an accepted margin of statistical uncertainty. In modern forensic age assessment, the reference populations are thus of enormous importance. It has been repeatedly emphasized by various authors that the quality of age assessment depends to a large extent on the quality and the applicability of the reference data and on how these data have been collected [20, 22–25].

In the past, various staging classifications have been presented for assessing third molar eruption in dental panoramic radiographs (PAN), which were more or less based on the fundamental stages of eruption described above [26–29]. These classifications vary in specific aspects, such as the choice of reference points or the level of detail in their descriptions. Our group recently demonstrated that the method proposed by Olze et al. in 2012 is particularly suitable for assessing the mandibular third molars’ eruption and should therefore be employed for further studies [29].

The aim of the present study was to generate new reference data for mandibular third molar eruption in a German population. The examinations were performed using the method by Olze et al. in 2012, which has been proposed to be most appropriate [27, 29]. Another aim of this study was to evaluate whether up-to-date reference data could demonstrate the suitability of the mandibular third molar eruption feature in PANs for determining the completion of the 18th year of age, which in many countries defines the age of majority.

## Material and method

The PANs used in this study were collected from a university dental clinic located in the north-western area of Germany (Münster, North Rhine-Westphalia region, Germany). The dental clinic consists of multiple specialized departments, thereby constituting a study population drawn from patients receiving care across various domains, including dental surgery, orthodontics, prosthodontics, and conservative dentistry. Importantly, all the PAN utilized in this study were originally acquired for medical purposes. The sample of PANs for this study was randomly selected for retrospective, blinded evaluation, stratified by each year of age between 15 and 26 years, which is in a line with comparable publications on the subject [26], with the age range intended to capture the entire range of development in mandibular third molar eruption.

Age was defined in annual increments (for example, “15 years” includes individuals aged between 15.00 and 15.99 years). Leap years were taken into account for the calculation of age. For inclusion in the study, age of the participants at the time of the X-ray examination had to be known beyond doubt. It can be assumed that the socioeconomic status of the study participants reflects the one of the catchment area (as the university dental clinic serves as the only tertiary care referral center in the region), which can be described as a high-income region.

With the intention of collecting valid reference values, we aimed to acquire a sample of 550 digital PANs, evenly distributed by each year of age and by sex. To accommodate subsequent exclusions of unratable PANs, the initial size of the sample was inflated to 660. The compilation of the X-ray images was conducted by personnel who was not involved in the subsequent examinations. The criteria for image acceptance in this first step were the appropriate age at the time of the radiographic examination and the presence of at least one mandibular third molar. Where multiple PANs existed for an individual, only the first eligible PAN was selected for study.

The actual inclusion and exclusion criteria were applied when the examiners viewed the 660 radiographs. The inclusion criteria stipulated the necessity for image quality that aligned with the research objectives. Individuals with displaced or retained mandibular third molars were also omitted from the study. The assessment of retentions adhered to established clinical criteria, with e.g. an angle exceeding 30 degrees in the mesio-distal direction being an exclusion criterion [30, 31]. In addition, all PANs indicating other pathologies such as bone fractures, cysts, carious lesions on the third molars, dental restorations on the third molars or orthodontic appliances on the third molars were also excluded.

**Fig. 1:**
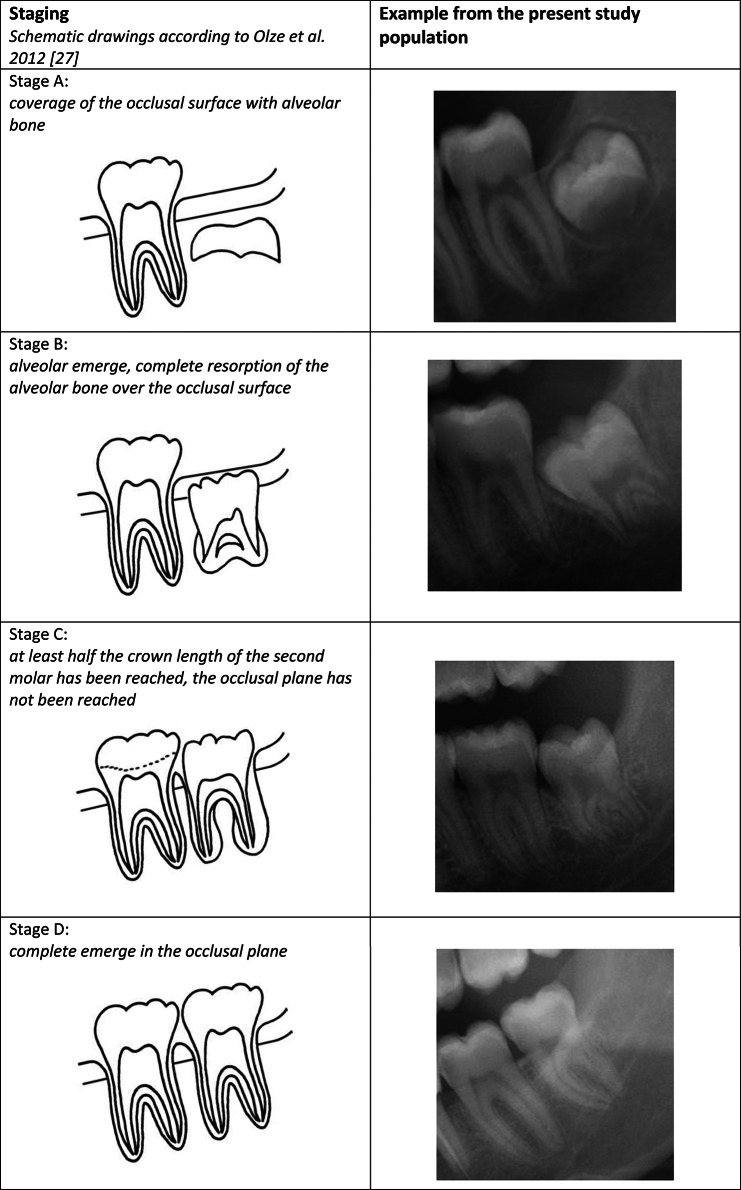
Schematic drawings according to Olze et al. (2012). Example from the present study population. *Original version of the schematic drawings: Olze A, Peschke C, Schulz R, Schmeling A (2012) [Application of a modified stage classification in evaluating wisdom tooth eruption in a German population]. Arch Kriminol 229:145–153*

The evaluations were performed according to the classification *by Olze et al. (2012)* [27] (Fig. 1):

A. Coverage of the occlusal surface with alveolar bone.
B. Alveolar emerge, complete resorption of the alveolar bone over the occlusal surface.
C. At least half the crown length of the second molar has been reached, the occlusal plane has not been reached.
D. Complete emerge in the occlusal plane.

Radiographs were assessed in DICOM format at appropriate workstations using synedra Personal View software version 22.0.0 (synedra information technologies GmbH, Innsbruck, Austria). The set-up conditions were comparable for both examiners. For the evaluations, the software’s magnification tool and the gray level adjustment tool were used. The examiners were two board-certified dentists, who had already dealt with the method in detail in the context of previous studies [29]. In addition to eruption, the mineralization status of the teeth was assessed for orientation purposes, at least in the case of completely erupted teeth. Both examiners evaluated the radiographs independently. Where the ratings were found to disagree, the two examiners reached a consensus through subsequent arbitration.

Data management and statistical analyses were performed in Stata, version 13.0 (Stata Corp LP, College Station, Texas, USA).

## Results

A total of 605 PANs were included in the study. Radiographs were from 302 females and 303 males, aged 15.04 (female) to 25.99 (male) years (Table 1). The radiographs were captured between February 2013 and April 2020.

**Table 1:** Age and sex distribution of the study population

Age	Males (*n*)	Females (*n*)	Total (*n*)
15	22	27	49
16	25	24	49
17	29	28	57
18	29	26	55
19	30	26	56
20	29	26	55
21	28	29	57
22	26	27	53
23	30	31	61
24	25	26	51
25	30	32	62
Total (n)	303	302	605

The most frequent reason for a tooth not being evaluable was the absence of the tooth, as radiographic identification of one mandibular third molar was sufficient for inclusion in the study. (Percentage of all non-evaluable teeth: tooth 38: 47.7%; tooth 48: 55.1%). The second most common cause was mesial angulation of the teeth according to the above criteria (38: 42.1%; 48: 39.6%). Distal angulation led to exclusion in only one case for both teeth. In one case, tooth 48 was angulated buccally, causing it to be excluded. Further reasons for exclusion were the absence of the second molar, the presence of pathologies on the relevant teeth and inadequate image quality.

The mean, median, and minimum age increased with each stage for both sexes. In both sexes, stage A was the only stage, in which the mean age was below 18 years, although there was considerable overlap in age between all stages. On average, males were younger for each stage of third molar development, reaching approximate parity with females only in stage D. Among females, the minimum ages at stage D for both third molars were 16.11 years for tooth 38 and 16.13 years for tooth 48. Among males, however, the minimum ages at stage D were approximately one year older with 17.34 years for tooth 38 and 17.10 years for tooth 48. Thus, the minimum ages for stage D, which corresponds to a completed eruption, are below 18 years of age for both teeth and both sexes. The maximum age for all stages, including stage A, is over 25 years for both teeth and both sexes, representing the upper age limit of the studied population (Tables 2, 3, 4 and 5).

**Table 2:** Descriptive measures for each stage for tooth 38 [FDI] in females. Age in years, rounded to second decimal place

Stage	*N*	Mean	SD	Median	LQ	UQ	Min	Max
A	45	17.59	2.97	16.47	15.55	18.41	15.04	25.92
B	67	19.66	2.89	19.36	17.30	21.50	15.09	25.93
C	47	20.97	2.86	20.36	18.55	23.08	15.43	25.92
D	102	22.60	2.43	22.94	21.01	24.68	16.11	25.98

SD: standard deviation. LQ: lower quartile. UQ: upper quartile. Teeth (N) missing in total to the number of participants (Table 1) were not present or had to be excluded

**Table 3:** Descriptive measures for each stage for tooth 48 [FDI] in females. Age in years, rounded to second decimal place

Stage	*N*	Mean	SD	Median	LQ	UQ	Min	Max
A	50	17.49	2.82	16.55	15.55	18.41	15.04	25.92
B	77	19.43	2.65	19.11	17.63	21.01	15.09	25.93
C	32	21.34	2.67	21.18	19.36	23.30	16.11	25.92
D	105	22.27	2.56	22.75	20.87	24.32	16.13	25.98

SD: standard deviation. LQ: lower quartile. UQ: upper quartile. Teeth (N) missing in total to the number of participants (Table 1) were not present or had to be excluded

**Table 4:** Descriptive measures for each stage for tooth 38 [FDI] in males. Age in years, rounded to second decimal place

Stage	*N*	Mean	SD	Median	LQ	UQ	Min	Max
A	28	16.94	2.43	16.20	15.57	16.80	15.07	25.64
B	66	18.91	2.47	18.38	17.05	20.05	15.27	25.59
C	32	20.35	2.71	18.86	18.10	23.57	17.03	24.62
D	102	22.29	2.31	22.42	20.24	23.78	17.34	25.95

SD: standard deviation. LQ: lower quartile. UQ: upper quartile. Teeth (N) missing in total to the number of participants (Table 1) were not present or had to be excluded

**Table 5:** Descriptive measures for each stage for tooth 48 [FDI] in males. Age in years, rounded to second decimal place SD: standard deviation. LQ: lower quartile. UQ: upper quartile. Teeth (N) missing in total to the number of participants (Table 1) were not present or had to be excluded

Stage	*N*	Mean	SD	Median	LQ	UQ	Min	Max
A	35	16.78	1.81	16.25	15.65	17.31	15.07	22.93
B	64	19.77	3.00	19.30	17.41	21.79	15.27	25.72
C	33	20.21	2.51	19.92	18.28	21.78	17.03	25.99
D	103	22.08	2.29	22.27	20.39	23.79	17.10	25.95

**Fig. 2:**
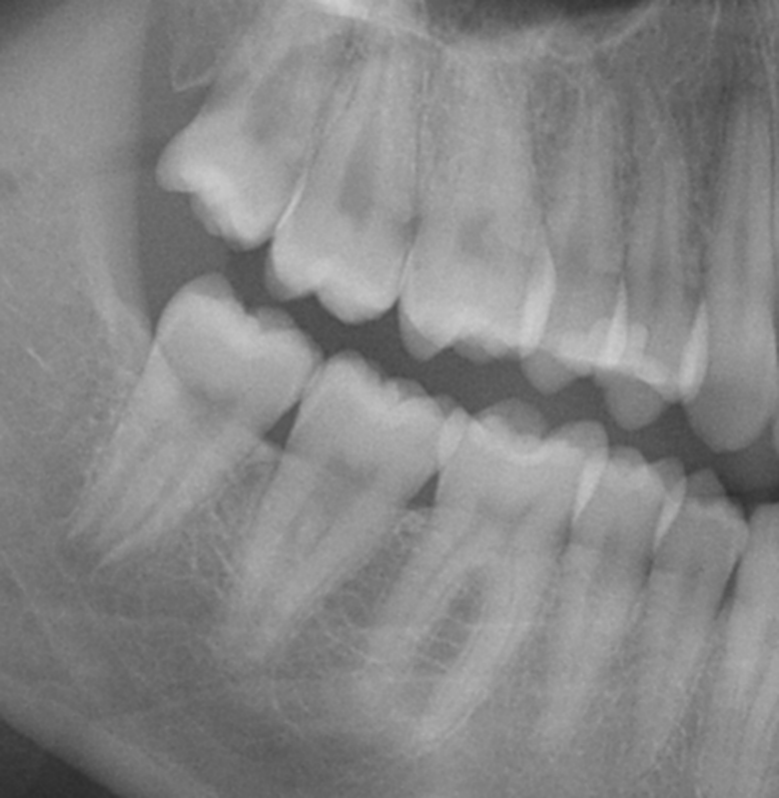
Right side section of the PAN of a 16.13-year-old female. Tooth 48 [FDI] with eruption stage D; complete eruption. Mineralization of the root at tooth 48 is not complete in the apical region. *For anonymization, the full PAN is not presented*

The youngest individual with stage D in both mandibular third molars, and thus a fully completed eruption, was a 16.13 year old female. In this case, the mineralization of the roots was not yet finished (Fig. 2). In all females younger than 17.5 years of age with at least one mandibular third molar having completed eruption (*n* = 9), mineralization of these teeth was not complete.

The youngest male, who had in both mandibular third molars stage D, was 17.34 years old. In this case as well, mineralization of the roots of both mandibular third molars was not yet complete. In the next youngest male, in whom both teeth were completely erupted (17.45 years), the teeth were completely mineralized.

Cases per stage range from 28 cases (stage A, tooth 38, males) to 105 cases (stage D, tooth 48, females). It is also shown that stage D was by far the most frequently detected in all teeth and both sexes (Tables 2, 3, 4 and 5).

## Discussion

The intention of the present study was to generate reference data for mandibular third molar eruption in PAN in a German population for forensic age assessment procedures. There are two applications for using the resulting reference data in practice.

First, the reference data can be used to estimate the most probable age of the person being examined. The distribution of age within each stage is known to be skewed. Therefore, information about the shape and distribution of age within each stage is best informed by quantiles, for which the median and lower and upper quartiles are useful summary statistics and the commonly presented for asymmetric distributions. To increase the accuracy of the prediction of the most probable age, different feature systems need to be combined [20]. However, issues of imprecision in determining the most probable age will remain due to biological variability and the spread of age-related features.

Second, the so-called “minimum age principle” can be used [8]: if exceeding legally relevant age limits is to be assessed with the highest level of certainty, the forensic minimum age of the person under examination is found by comparing the stage of the age-related feature to the reference data and deriving the age from the minimum age of the stage for the given age-related trait.

In practice, more than one feature system is used for age assessment [20]; in this case the highest minimum age found across the different features is considered to be the minimum age for the individual examined.

Due to this context and the importance of this principle, the minimum ages in the stages are critical. Since in practice the question of the age of majority of the examined subject often has to be clarified, it is necessary to determine whether the minimum age in a stage is above the age of 18 years. This was not the case, not even for the highest stage, D. Thus, the assessment of the eruption of mandibular third molars according to the minimum age principle is not suitable for proving information on majority (seen here as 18 years or older) based on our results.

As the accuracy of age assessment is heavily dependent on the quality of the underlying reference studies [22–24], guidelines for reference studies have been proposed [20]. The requirements formulated by Schmeling et al. in 2008 served as the basis for the current study [20]. Overall, we were able to meet these requirements. In particular, the distribution of age in our sample was approximately uniform [22]. Minor deviations between age groups in our population can be attributed to the practical conditions of the study such as the absence of a specific pre-selection of the X-ray images before the actual evaluation. In addition, we examined the biological sexes separately, as required, and provided critical information on the population studied.

Another requirement is for an adequate sample size [20]. This is particularly important with regard to the representation of the minimum age for a stage. For this purpose, a sufficient number of individuals with an age around the potential minimum age of a feature expression should be included. In a recent study from 2023, Sgheiza and Liversidge were able to demonstrate that sample size is the decisive aspect for the quality of reference studies in age assessment [24]. The study explicitly addressed dental age assessment [24]. Sheiza and Liversidge even gave specific numbers for filling the individual age groups. They stated a minimum population of 20–40 individuals per age group as a requirement [24]. Specifically, sample sizes of *n* < 20 per age group led to poorer age prediction results [24]. In this regard, it can be stated that our study meets this requirement for each age group within each sex.

The size of the reference sample also raises the question of the process of acquiring the data. This is commonly a challenge for studies on forensic age assessment, as previously deliberated by Roberts and Lucas in 2021, since assembling prospectively randomly selected population-based study populations is not feasible due to ethical constraints associated with X-ray examinations and radiation exposure [25]. Consequently, studies on forensic age assessment typically rely on X-ray images acquired for medical purposes, a practice that was also adhered to in our study [25]. It is assumed that samples consisting of orthodontic patients and young adults with wisdom tooth issues generally exhibit normal developmental patterns relevant to the research question [25]. However, it is noteworthy that Roberts and Lucas in 2021 underscore the necessity of excluding individuals displaying clinical and/or pathological irregularities from the examinations [25]. Our study adopted the principles proposed by Roberts and Lucas, leading to stringent exclusion criteria.

Olze et al. examined a total of 666 PANs from 144 males and 522 females, aged 12–26 years, within a German population in 2008 [26]. The staging classification Olze et al. developed in 2008 was highly akin to the method of our study, with the exception of Stage C, which varies from the method used in our study. Concerning the complete eruption of mandibular third molars, it is notable that Olze et al. reported a minimum age of 19.5 years for males, while for females this age was 17.4 years, which were considerably higher than the corresponding values in our study. This is particularly important for males, as the minimum age from the 2008 Olze study was above the age limit of 18 years. Our results show that the eruption can be completed before the age of 18 years in males in the population studied. One possible explanation for this difference is that Olze et al. included only one individual in the male 17 years age group, and consequently Olze et al. may have missed complete eruption at this age because of the small number of individuals in this age group. In contrast, Olze et al. were able to collect 45 individuals in the 16 years old age group in females. This group was larger than in our study. Nevertheless, Olze et al. did not find a complete eruption in this age group, in contrast to our study. Similar to the results of our study, Olze et al. also found an earlier completion of the eruption in females.

In 2012, Olze et al. again studied the eruption in a German population. In this study, the staging also used in our study was applied [27]. Olze et al. examined a total of 606 PANs from 515 females and 91 males aged 12 to 25 years [27]. The minimum ages for stage D were now 20.0 (tooth 38) and 15.2 (tooth 48) years for the females. The comparable minimum ages for male teeth 38 and 48 were 20.6 and 20.6 years, respectively. Here, the minimum age of 15.2 years at tooth 48 for females is noteworthy. This value is almost exactly one year lower than the value found in our study for the minimum age of complete eruption in females. A remarkable aspect of this study is that Olze et al. in 2012 did not include a single 17 year old male. Thus, they could not find a completed eruption in this age group with their study. The male age groups at 18 and 19 years were also relatively small with only five individuals in each group. In contrast, there were more females in the 15 years old age group (*n* = 48) than in our study.

In 2019, Gambier et al. examined eruption in a French population comprising a total of 557 individuals (340 males, 217 females) using PAN [32]. Despite the utilization of a different staging system than the ones by Olze et al. from 2008 to 2012, the results were compared to our study given the geographical proximity and apparently comparable socio-economic status of the populations. The maximal stage of Gambier et al.’s study, which also corresponds to complete eruption, is comparable to stage D in our study. Gambier et al. identified a minimum age of 15.11 years for males and 15.28 years for females for full eruption. In the 15 years old age group, Gambier et al. included 22 females and 17 males. Gambier et al. concluded that eruption was not a suitable indicator for the 18 years age limit. The minimum ages for complete eruption found by Gambier et al. are well below the results of our study. It is striking that the result for females is comparable to the result of the 2012 study by Olze et al. [27]. Gambier et al. stated that primary retained teeth were excluded from their study. They did not provide more detailed information on the criteria of retention or angulation of the teeth.

Future studies should validate the completion of mandibular third molar eruption by age 15 in relevant populations, as our study did not contribute to the validation of this aspect.

In the past, the question of the extent to which eruption is linked to ethnicity has been raised repeatedly [33–42]. Specifically, it was discussed that specific ethnic groups could pass through the individual stages of eruption earlier than others [43]. In instances marked by substantial interethnic disparities, the utilization of a standard reference would result in inaccurate and potentially spurious estimations. Existing results suggest that individuals of African descent exhibit an accelerated eruption [42], while Asians, in comparison to Europeans, demonstrate delayed eruption [43]. The basis for these investigations were populations that did not entirely meet the requirements for reference populations [43]. It remains pending whether the results can be substantiated in further, high-quality reference studies. Therefore, high-quality studies in different ethnic groups, following the requirements for reference studies and conducted with a comparable method, are urgently needed.

Our results demonstrate that eruption in a German population may be fully completed by the age of 16 or 17 years. The practical usefulness of the method for the proof of majority is thus limited. However, it is important to note that our results indicate that the development of mandibular third molars may not be complete, even if the teeth are fully erupted (Fig. 2). This finding is in accordance with the existing literature [21, 44]. Because mandibular third molars may not be fully developed after eruption, the assessment of mandibular third molar mineralisation may yet offer an alternative means for DAE.

Nevertheless, our reference data may offer a means of estimating the most probable age of a person based on mandibular third molar eruption, ideally in combination with other age-related traits. Therefore, determination of eruption should continue to be an important component of forensic age assessment.

## Conclusion

Contrary to previously published studies of German populations, the eruption of mandibular third molars may be completed before the age of 18 years, particularly among males. Therefore, the feature of mandibular third molar eruption alone is not suitable for confirming this legally significant age threshold. In the future, high-quality reference studies should investigate the eruption of mandibular third molars in various ethnic groups.

## Data Availability

The datasets generated during the current study are available from the corresponding author on reasonable request.
